# EHMTI-0291. Chronic headache is associated with mental vulnerability, depression, and neuroticism and poor mental health-related quality of life: a cross-sectional population study

**DOI:** 10.1186/1129-2377-15-S1-B2

**Published:** 2014-09-18

**Authors:** S Ashina, AC Lyngberg, L Bendtsen, D Buse, RB Lipton, R Jensen

**Affiliations:** 1 Neurology, Mount Sinai Beth Israel Icahn School of Medicine at Mount Sinai, New York, USA; 2 Unit for Quality and Patient Safety, Capital Region of Denmark, Hvidovre, Denmark; 3 Neurology, Danish Headache Center University of Copenhagen Glostrup Hospital, Glostrup, Denmark; 4 Neurology, Montefiore Headache Center Albert Einstein College of Medicine, New York, USA

## Introduction

Psychiatric comorbidity in migraine and tension-type headache (TTH) is well established.

## Aim

To study differences in mental health variables in relation to headache subtype and frequency.

## Methods

A sample of 547 subjects completed a questionnaire based on ICHD-1 and provided data on mental vulnerability (12-item scale), depression (MDI), neuroticism (Eysenck Personality Questionnaire) and mental health-related quality of life (SF-12). Results were adjusted for age, gender and education in a multiple regression model. Chronic headache indicated headache on ≥15 days per month.

## Results

Mental vulnerability scores (mean ± SD) were highest for chronic headache (migraine +/- TTH) headache (6.1 ± 1.9), followed by chronic TTH (5.7 ± 2.1), episodic migraine +/- TTH (4.2 ± 1.9), episodic TTH (4.2 ± 1.6), no headache (3.1 ± 1.4),(p<0.001). Neuroticism scores were highest for chronic TTH (11.7 ± 8.2), followed by chronic headache (9.5 ± 4.9), episodic TTH (8.9 ± 4.9), episodic migraine +/- TTH (8.8 ± 4.5), no headache (6.2 ± 4.3),(p<0.001). Depression scores were highest for chronic headache (13.8 ± 10.4), followed by chronic TTH (12.6 ± 14.0), episodic migraine +/- TTH (7.4 ± 6.3), episodic TTH (7.2 ± 7.3), and no headache (4.4 ± 4.9),(p<0.001). SF-12 scores were lowest in chronic TTH (43.0 ± 11.6), followed by chronic headache (48.1 ± 10.1), episodic TTH (51.2 ± 8.8), episodic migraine +/- TTH (50.4 ± 8.0), no headache (53.9 ± 6.8),(p<0.001).

## Conclusions

Chronic migraine and TTH areis associated with low mental health-related quality of life, high mental vulnerability, depression and neuroticism scores.

